# The Effects of Thiamine Tetrahydrofurfuryl Disulfide on Physiological Adaption and Exercise Performance Improvement

**DOI:** 10.3390/nu10070851

**Published:** 2018-06-29

**Authors:** Wen-Ching Huang, Hui-Yu Huang, Yi-Ju Hsu, Wan-Hsiung Su, Sih-Yu Shen, Mon-Chien Lee, Che-Li Lin, Chi-Chang Huang

**Affiliations:** 1 Department of Exercise and Health Science, National Taipei University of Nursing and Health Sciences, Taipei 11219, Taiwan; wenching@ntunhs.edu.tw; 2 Department of Food Science, Nutrition, and Nutraceutical Biotechnology, Shih Chien University, Taipei 10462, Taiwan; maggieh@mail.usc.edu.tw; 3 Graduate Institute of Sports Science, National Taiwan Sport University, Taoyuan 33301, Taiwan; 1041302@ntsu.edu.tw; 4 Prince Pharmaceutical Co., Ltd., New Taipei 24141, Taiwan; wssu@prince-pharm.com.tw (W.-H.S.); syshen@prince-pharm.com.tw (S.-Y.S.); 5 Department of Orthopedic Surgery, Shuang Ho Hospital, Taipei Medical University, New Taipei City 23561, Taiwan; 6 Graduate Institute of Metabolism and Obesity Sciences, Taipei Medical University, Taipei City 11031, Taiwan

**Keywords:** thiamine, derivatives, fatigue, safety, physiological adaption

## Abstract

Thiamine, named as vitamin B1, is an important cofactor for the critical enzymes regarding to glucose metabolism, like transketolase, pyruvate dehydrogenase, and alpha-ketoglutarate dehydrogenase. The thiamine tetrahydrofurfuryl disulfide (TTFD) is a derivative of thiamine with higher bioavailability and solubility than thiamine and has been widely applied to health maintenance and disease therapy. Higher physical activities are associated with higher thiamine supplements for efficient energy metabolism. Furthermore, the effective dose of TTFD, beneficial to exercise physiological adaption and performance, still be further validated and the safety evaluation were also an important issue to be considered for potential application. ICR (Institute of Cancer Research) strain mice were allocated as 0, 50, 100, and 500 mg/kg dose groups and administrated by oral gavage consecutively for 6 weeks. Physical activities including grip strength and aerobic endurance were measured. Various fatigue-associated biochemical variables such as lactate, glucose, blood urine nitrogen (BUN) or creatine kinase (CK), were also assessed. The levels of liver and muscle glycogen were measured as an indicator of energy storage at the end of the experiment. Toxicity assessments for long-term supplementation were also further evaluated for safety consideration. TTFD supplementation significantly increased the endurance and grip strength and demonstrated beneficial effects on lactate production and clearance rate after an acute exercise challenge. The TTFD supplementation significantly mitigated the BUN and CK indexes after extended exercise and elevated the glycogen content in the liver and muscle tissues. According to body composition, biochemical and histopathological data, daily administration of TTFD for over 6 weeks (subacute toxicity) also demonstrated reasonable safety results for long-term and adequate supplementation. The toxicity of TTFD were also considered as safety for long-term supplementation with indicated doses. Furthermore, the TTDF could be applied to not only the health promotion but also improvement of exercise physiological adaption and the TTFD could be further considered as potential ergogenic aids combined with different nutrient strategy.

## 1. Introduction

Thiamine, also known as vitamin B1, exists in a variety of thiamine-rich food sources including beef, liver, dried milk, oats, pork, eggs, seeds, legumes, peas and fortified grain and it is vitally important to maintain normal body function. Thiamine could play an important role in the nervous system [1,2], heart health [3], energy metabolism [4], and psychological health [5]. A recommended dietary allowance (RDA) has been announced by the U.S. Food and Nutrition Board for vitamin B1 (thiamin) to prevent the deficiency in a healthy population (Food and Nutrition Board). The RDA could be calculated by calorie intake referring to 0.5 mg per 1000 kcal and the intake range could be 1.0–1.1 mg per day for women and 1.2–1.5 mg for men, referring to an average caloric intake. The specific population also showed inadequate dietary intake and thiamin insufficiency so the RDA could be increased to 1.5 mg/day for health maintenance [6]. Physical activity could also affect the thiamine requirement in addition to gender, age, and physiological status [7]. Thiamine could be converted to three phosphorylated forms, thiamine monophosphate (TMP), thiamine pyrophosphate (TPP), and thiamine triphosphate (TTP), after intestinal absorption, but the rate-limiting transport system for thiamine absorption causes a bioavailability issue [8].

Physiological fatigue is caused by inadequate rest, physical effort or mental strain and could be categorized as peripheral fatigue, produced by changes at or distal to the neuromuscular junction. Central fatigue originates at the central nervous system (CNS), which decreases the neural drive to muscles. Fatigue development could result from different factors including metabolites, energy demands, oxygen availability, ion regulation, neural contributions, and physiological reactants during contractive processes [9].

The biomarker applied to assess fatigue could be affected by exercise types, contraction type, durations, and fatigue degree. Three categories, namely, energy metabolism, oxidative stress, and inflammatory indexes, are usually assessed according to the mechanism and metabolic changes during muscle fatigue [10]. Exercise fatigue could successfully be induced by treadmills and swimming methods and the exercise performance and physiological indexes could be easily assessed [11,12]. However, the treadmill is generally equipped with an electric shock stimulator to enforce continued exercise and the shock stimulation could possibly interfere with the quantitation of running endurance, as well as confound measurements of post-exercise serum hormone and cytokine levels [13]. The swimming test could ensure a higher distinguished capacity with stronger survival instinct as compared to the treadmill [14,15].

In previous studies, different types of supplements were reported for potential nutrient supplements including nutritional supplements, synthetic and natural products to mitigate exercise fatigue by the above-mentioned platforms [9]. Energy efficient supply could be considered as an important factor to maintain the performance and postpone the onset of fatigue. The active form of thiamine (TPP) serves as a cofactor for the critical enzymes involved in glucose metabolism, such as transketolase, pyruvate dehydrogenase, and alpha-ketoglutarate dehydrogenase [16]. In perspective of biochemical structure, thiamine consists of a pyridinium ring joined to a thiazolium ring by a methylene bridge. The derivative, TTFD, is synthesized by attaching a mercaptan to the sulfur atom of the thiazolium ring. The mercaptan, left outside the cell membrane as the complete molecule, is hydrolyzed to deliver thiamine into the cell and has been well studied for its metabolic breakdown [17]. This transit through cell membranes provides a high concentration of the vitamin in the cell and the reaction does not require a transport system to assist in intracellular absorption of thiamine [18]. The bioavailability is significantly higher than thiamine salt in a variety of organs [19]. The daily thiamine supplement is positively associated with physical activities in the announcement of nutrient and energy intakes for the European Community but related studies did not provide sufficient evidence to address the effective doses and physiological adaption.

The RDA of thiamine is a relatively low dose to maintain the physiological functions from food source or pharmaceutical supplement. Thus, whether the higher thiamine supplementation could be beneficial to specific populations, athletes and blue-collar workers, is an interesting topic to the purpose of health promotion. In the current study, we applied the thiamine derivative, TTFD, to our well-established animal models to investigate the effects of physiological adaptation and physical activities. In addition, the toxic observation with the relative high doses to clinical application was also an important issue that requires further investigations.

## 2. Materials and Methods

### 2.1. Materials

**Figure 1:**
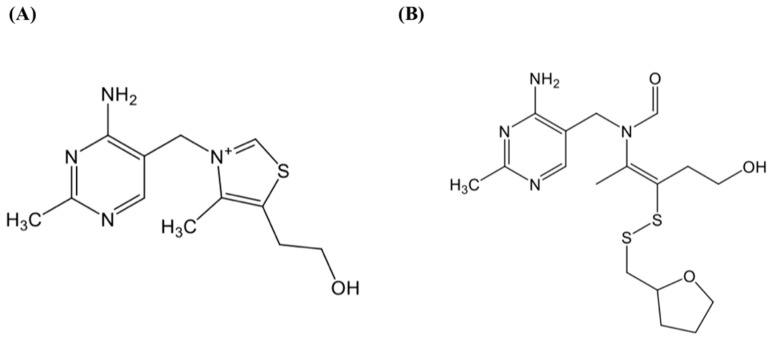
The structure of thiamine (**A**) and thiamine tetrahydrofurfuryl disulfide (**B**).

The supplement, thiamine tetrahydrofurfuryl disulfide (TTFD), provided by Prince Pharmaceutical Co., Ltd. (New Taipei City, Taiwan) was used in the current study to investigate exercise physiological efficacy. The chemical structures of thiamine and TTFD are demonstrated in Figure 1 and the appearance of TTFD is a white power with improved solubility and bioavailability.

### 2.2. Animals and Experiment Design

Male ICR (Institute of Cancer Research) mice (6 weeks old, SPF level) purchased from BioLASCO Taiwan (Yi-Lan, Taiwan) were usd in the current study. The ICR mice was an outbred mouse species and have been widely used in many fields including toxicity, pharmacology, drug efficacy, and immunology. The animals were acclimatized and allowed food ad libitum for 1 week prior to the experiments. The standard laboratory diet # 5001 (PMI Nutrition International, Brentwood, MO, USA) and distilled water were provided to animal ad libitum, and the environment was maintained in constant photoperiod, humidity, and temperature (12 h light/12 h dark cycle, 55–65%, and 24 ± 2 °C, respectively). The Institutional Animal Care and Use Committee (IACUC) of National Taiwan Sport University approved all animal experiments in this study, and the study conformed to the guidelines of protocol IACUC-10619 approved by the IACUC ethics committee.

**Figure 2:**
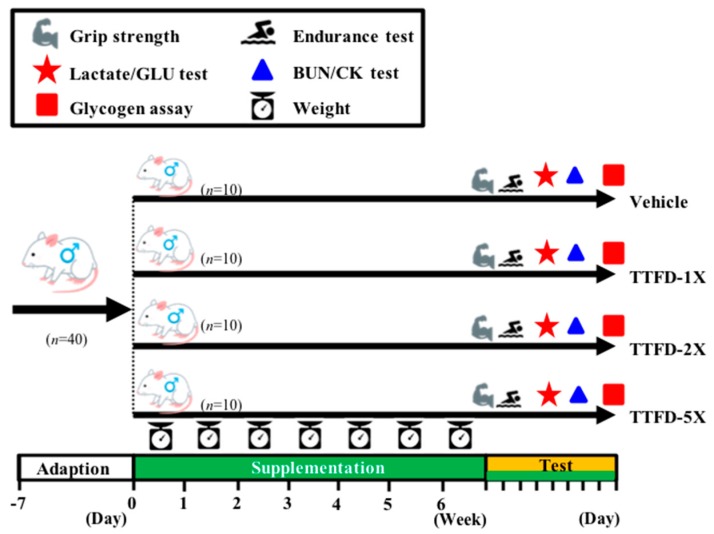
Experimental designs for the effects of thiamine tetrahydrofurfuryl disulfide (TTFD) on exercise adaption. The animals were randomly assigned to the indicated four groups (Vehicle, TTFD-1X, TTFD-2X, and TTFD-5X) and consecutively supplemented the TTFD until the end of the experiments. The physical capacities and related biochemistries were assessed within test duration.

The administration of TTFD dosage was studied from 50 mg/kg to 340 mg/kg body weight with different biological efficacies in previous animal references [19,20,21]. The detailed experimental procedure is illustrated in Figure 2. All animals were given an acclimation period to adapt to the environment and diet. The weight, dietary, and social behaviors were monitored during the supplementation period and the daily supplementation began at regular times with freshly prepared TFFD solutions. Treatment dosages of 0, 100, 200, and 500 mg/kg/day were designated as vehicle, TFFD-1X, TFFD-2X, and TFFD-5X and were administrated by oral gavage with a volume 10 mL/kg BW. Physical fitness was evaluated by grip strength and aerobic endurance capacities and the exercise-related biochemistry was immediately assessed after a fixed exercise time/intensity.

### 2.3. Exercise Endurance Performance Test

Exercise performance was based on the survival motives to assess the aerobic endurance capacities. The animals were loaded with a weight equivalent to 5% individual body weight and forced to swim in a tank until exhaustion. The persistent time from beginning to exhaustion was recorded as endurance index. The detailed procedures and protocol were described in our previous article [22].

### 2.4. Forelimb Grip Strength Test

A low-force testing system (Model-RX-5, Aikoh Engineering, Nagoya, Japan) for forelimb grip strength was used to measure the grip strength. The details have been described previously [23].

### 2.5. Determination of Fatigue-Associated Biochemical Variable

The effect of TFFD supplementation on fatigue-associated biochemical indexes was slightly modified to accurately demonstrate the physiological status based on our previous reports [24]. The fatigue-associated variables were assessed with fasted status to reflect the real physiological adaption under exercise interventions. For the lactate and glucose profile, the blood sampling time points were pre-exercise, immediately after 10 min acute exercise, and then after 20 min rest. The other indexes, such as blood urine nitrogen (BUN) and creatine kinase (CK), were immediately assessed at the 60 min rest time point after 90 min of extended exercise. The blood samples with fully coagulation were centrifuged at 1000× *g* and 4 °C for 15 min for serum separation and determined by use of an autoanalyzer (Hitachi 7060, Hitachi, Tokyo, Japan).

### 2.6. Clinical Biochemical Profiles

TTFD supplementation was consecutively administrated for 6 weeks until animal sacrifice. All mice were euthanatized by 95% CO_2_ asphyxiation one hour after the last treatment and immediately sampled blood by cardiac puncture. Serum was separated by centrifugation and clinical biochemical variables, including aspartate aminotransferase (AST), alanine transaminase (ALT), ammonia (NH_3_), creatine kinase (CK), glucose (GLU), blood urea nitrogen (BUN), creatinine (CREA), uric acid (UA), total cholesterol (TC), triglycerides (TG), albumin (ALB), and total protein (TP) were measured by use of an autoanalyzer (Hitachi 7060).

### 2.7. Body Composition and Glycogen Content Analysis

After sacrifice, the important visceral organs, including liver, kidney, heart, lung, muscle, epididymal fat pad, and brown adipose tissue, were accurately excised and weighed. Then, the organs were saved into 10% formalin for further histopathology. Part of muscle and liver tissues was stored in liquid nitrogen for glycogen content analysis as described previously [24].

### 2.8. Histopathology

The visceral organs preserved in 10% formalin were trimmed and embedded in paraffin for tissue sections with 4 μm thickness slices. Tissue sections were further stained with hematoxylin and eosin (H&E) and examined under a light microscope equipped with a CCD camera (BX-51, Olympus, Tokyo, Japan) by a veterinary pathologist.

### 2.9. Statistical Analysis

The data were represented as mean ± SEM. The statistical difference among groups in physical activities, biochemistry, lactate, body weight, body composition, dietary, and glycogen content were analyzed by one-way analysis of variance (ANOVA) and the two-way mixed ANOVA was also applied to lactate and growth curve profile. The dose-effect trend analysis was further analyzed by the Cochran-Armitage test with SAS v. 9.0 (SAS Inst., Cary, NC, USA). Data were considered statistically significant when the probability of a type I error was less than 0.05.

## 3. Results

### 3.1. The Effects of TTFD Supplementation on Endurance Capacity

**Figure 3:**
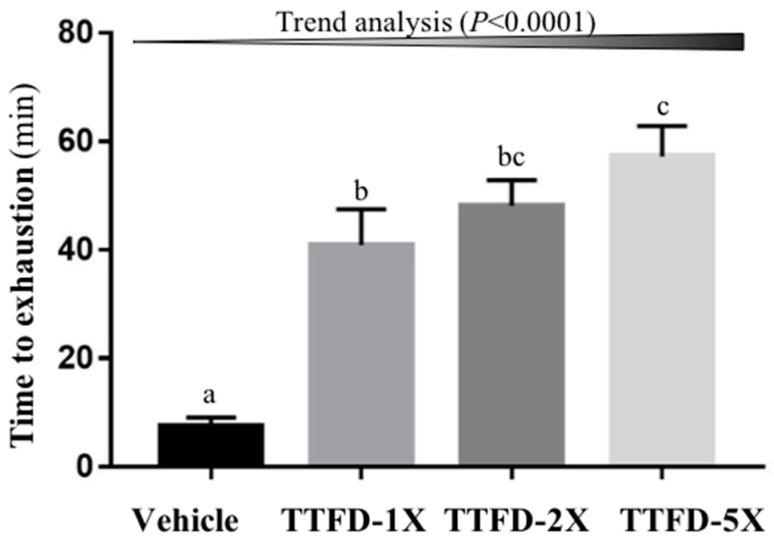
Effect of 4-week TTFD supplementation on exhaustive swimming time. Data are mean ± SEM for *n* = 10 mice per group. Columns with different letters (a, b, c) are significantly different at *P* < 0.05.

The endurance capacity was measured by an exhaustive swimming test and it showed a significant difference among groups (*F* (3, 36) = 19.91, *P* < 0.001). The TTFD supplementation groups (TTFD-1X, TTFD-2X, and TTFD-5X) were significantly higher than the vehicle group showing a 5.4-, 6.4-, and 7.6-fold increase and demonstrated significant dose-dependent effects (*P* < 0.001) in trend analysis (Figure 3).

### 3.2. The Effects of TTFD Supplementation on Grip Strength

**Figure 4:**
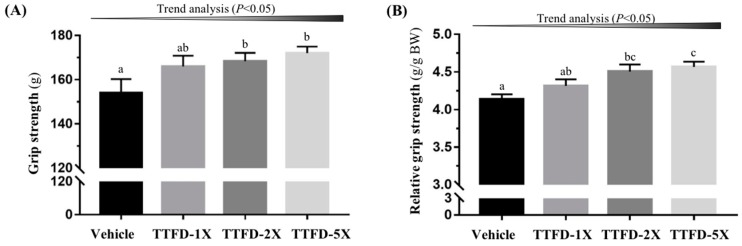
Effect of 4-week TTFD supplementation on absolute forelimb grip strength (**A**) and forelimb grip strength (%) relative to body weight (**B**). Data are mean ± SEM for *n* = 10 mice per group. Columns with different letters (a, b, c) are significantly different at *P* < 0.05.

The grip strength showed significant differences among groups in absolute strength (*F* (3, 36) = 2.85, *P* = 0.045) and relative strength calibrated by individual body weight (*F* (3, 36) = 6.03, *P* = 0.003). The TTFD supplementation groups (TTFD-2X, and TTFD-5X) were significantly higher than the vehicle group (*P* <0.05) with regard to absolute strength (1.09-, and 1.11-fold, respectively) and relative strength (1.08-, and 1.10-fold, respectively). Both grip strength measures also showed significant TTFD dose-dependent effects (*P* < 0.05) in the trend analysis (Figure 4).

### 3.3. The Effects of TTFD Supplementation on Exercise-Related Biochemical Indexes after Exercise Challenge

The lactate metabolite which is highly associated with exercise physiological status was assessed pre-exercise, immediately post-exercise, and after rest (three time points) with the TTFD treatments (Table 1). It showed significance difference in the supplementation main effect (*F* (3, 36) = 8.04, *P* = 0.001) and time main effect (*F* (2, 72) = 123.4, *P* < 0.001). The interaction effect was also significant (*F* (6, 72) = 3.365, *P* = 0.007). Further analysis of the three time points showed a significant difference in the post-exercise (*F* (3, 36) = 8.11, *P* < 0.001) and after rest (*F* (3, 36) = 5.24, *P* = 0.005) time points. The vehicle group was significantly higher than other TTFD treatment groups (TTFD-2X, and TTFD-5X) at time points post-exercise and after rest by one-way ANOVA. In the self-comparison indexes, lactate production demonstrated significant beneficial effects with TTFD-5X supplementation (*F* (3, 36) = 2.79, *P* = 0.048) but there was no significant difference in clearance rate among groups (*F* (3, 36) = 0.174, *P* = 0.913).

**Figure 5:**
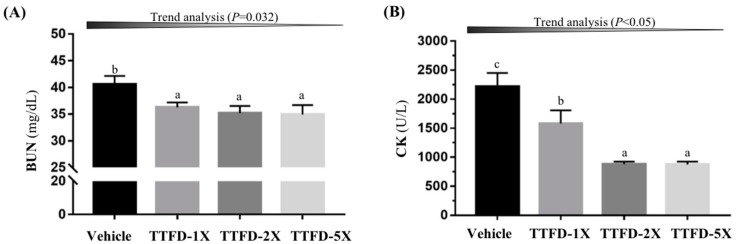
Effect of 4-week TTFD supplementation on the serum BUN (**A**) and CK (**B**) levels after extended exercise challenge. The indicated four groups underwent 90 min swimming exercise and blood was sampled after 60 min rest. Data are mean ± SEM for *n* = 10 mice per group and the columns with different letters (a, b) are significantly different at *P* < 0.05.

The other metabolic indicator, BUN, demonstrated significance after extended exercise (*F* (3, 36) = 3.34, *P* = 0.033) (Figure 5A). TTFD supplementation (TTFD-1X, TTFD-2X, and TTFD-5X) significantly decreased the exercise-induced BUN elevation by 10–14% (*P* < 0.05) showing a dose-dependent trend (*P* = 0.032). The other important injury index, CK, also showed significant difference among groups (*F* (3, 36) = 15.02, *P* < 0.001) (Figure 5B). The TTFD supplementation could mitigate the CK increase by 28.6, 60.2, and 60.4%, respectively, and showed a dose-dependent trend (*P* < 0.0001).

### 3.4. The Effects of TTFD Supplementation on Energy Metabolism

**Figure 6:**
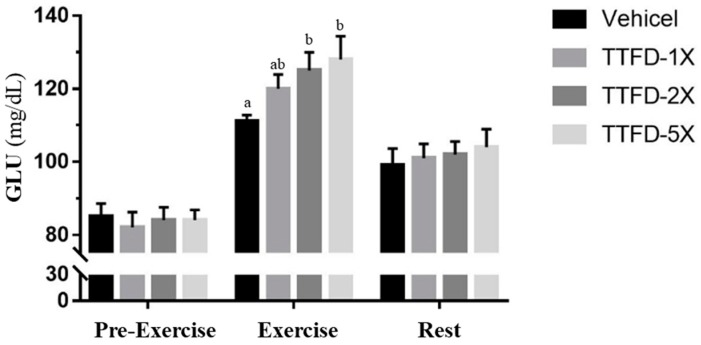
Effect of 4-week TTFD supplementation on the serum GLU levels profile during exercise. The indicated four groups collected three time points including beginning, 10 min acute exercise and 20 min rest. Data are mean ± SEM for *n* = 10 mice per group and the columns with different letters (a, b) are significantly different at *P* < 0.05.

Glucose is the most important fuel for energy demands and supply during exercise. The fasted glucose levels before exercise did not show a significant difference amount groups. The TTFD groups (TTFD-2X, and TTFD-5X) exhibited significantly higher levels than the vehicle group (*F* (3, 36) = 3.12, *P* = 0.042) immediately after fixed exercise time/intensity but there was no significant difference in the rest time point after 20 minutes among groups (*F* (3, 36) = 0.231, *P* = 0.874) (Figure 6).

### 3.5. The Effects of TTFD Supplementation on Glycogen Content

**Figure 7:**
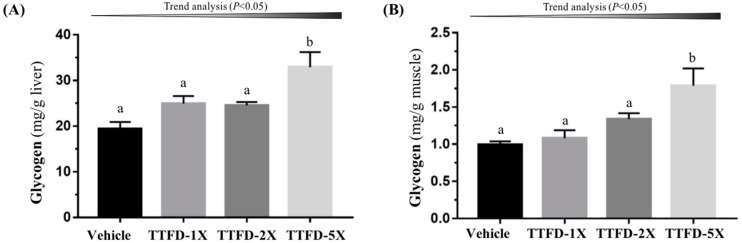
Effect of 4-week TTFD supplementation on hepatic (**A**) and muscle (**B**) glycogen levels. Data are mean ± SEM for *n* = 10 mice per group. Bars with different letters (a, b) are significantly different at *P* < 0.05.

Glycogen is mainly stored in the liver and muscle tissue with the purpose of energy supply and homeostasis. TTFD supplementation was shown to affect glycogen content in the liver (Figure 5A; *F* (3, 36) = 7.47, *P* = 0.001) and muscle (Figure 5B; *F* (3, 36) = 6.79, *P* = 0.001). Hepatic and muscular glycogen content with TTFD-5X supplementation significantly increased as compared to the other groups (vehicle, TTFD-1X, and TTFD-2X) (Figure 7). Both hepatic and muscular glycogen showed significant dose-dependent trends (*P* < 0.05).

### 3.6. Subacute Oral Toxicity Evaluation of TTFD Supplementation

Subacute toxicity, following OECD Guideline 407, was performed to evaluate the safety of the TFFD supplementation with relative high doses to humans. Several parameters including behavior, diet, growth curve, organ weight, histopathology and biochemical variables were evaluated for the subacute toxic effects of TTFD supplementation. No abnormal behavior was observed among the groups with daily TTFD administration. As shown in Table 2, the main effect (supplementation) did not significantly show any difference (*F* (3, 36) = 0.43, *P* = 0.732) but the main effect (time) showed a significant difference (*F* (5, 140) = 209.7, *P* < 0.0001) which indicated time-dependent growth. However, the interaction effect (supplement × time) was not significantly different (*F* (15, 140) = 0.702, *P* = 0.683) and further analysis at different time points showed no significant differences among groups. Diet and water intake also did not significantly differ among groups with dose-dependent TTFD supplementation (*F* (3, 120) = 0.003, *P* = 0.949; *F* (3, 120) = 0.018, *P* = 0.986).

Body composition may also reflect the effect of supplementation on different organs. As shown in Table 3, the weight of live, muscle, kidney, heart, lung, epididymal fat pad, and brown adipose tissue was not significantly different among groups (*P* > 0.05) and the relative organ weight adjusted by individual weight also showed the same statistical results (*P* > 0.05). This demonstrated that TTFD supplementation do not have an impact on different organs with continuous administration. Clinical biochemistry was also used to evaluate the effects of supplementation on physiological status. As shown in Table 4, TC showed a significant difference among groups (*F* (3, 36) = 3.41, *P* = 0.031) and TTFD treatments (TTFD-2X, and TTFD-5X) were significantly lower than the vehicle groups. The other indexes including hepatic function, blood lipid, renal function, injury and energy metabolites were not significantly different.

### 3.7. Histopathological Observation

**Figure 8:**
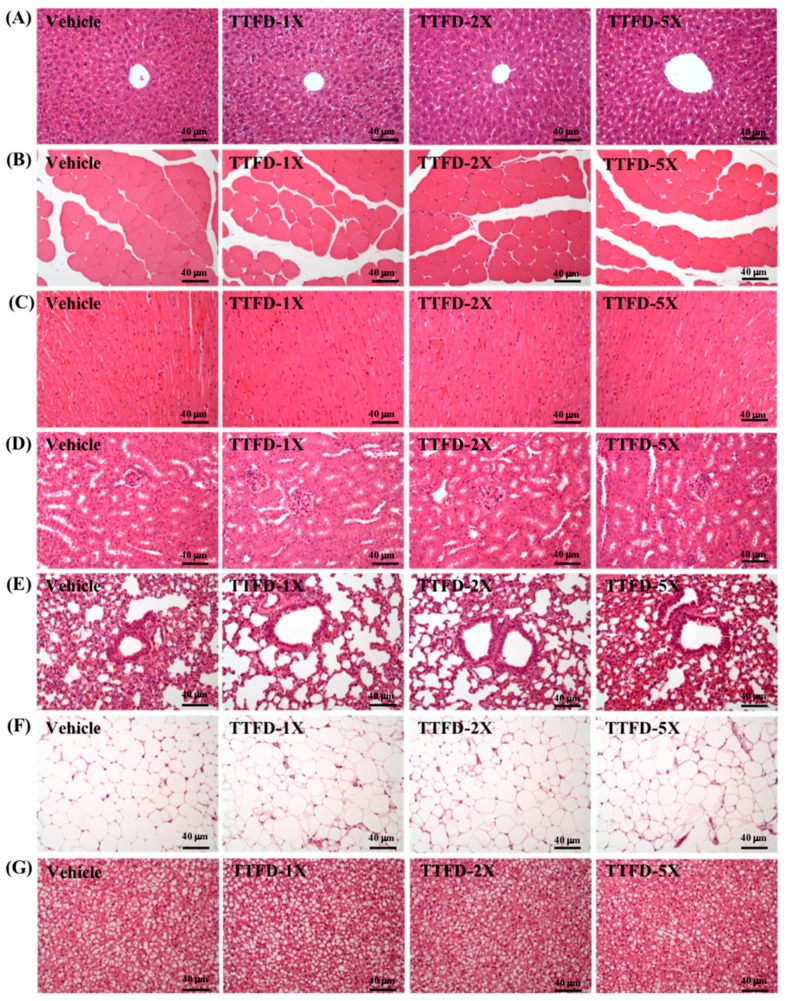
Effect of TTFD supplementation on histomorphologic features of the liver (**A**), muscle (**B**), heart (**C**), kidney (**D**), lung (**E**), white fat tissue (**F**) and BAT tissue (**G**) in mice. Specimens were photographed under a light microscope. (H&E stain, magnification: 200×; bar, 20 μm).

As shown in Figure 8, histological examinations of the main organs including liver, muscle, heart, kidney, lung, epididymal fat pad, and brown adipose tissue were performed at the end of the study period. The figure shows representative photomicrographs of organs from the mice of vehicle and indicated dosages of TTFD treated groups. HE staining of the liver showed normal hepatic architecture such as hepatocytes, bile duct and sinusoid. Histological observation of the kidney showed a normal renal architecture with a normal appearance of glomerulus, tubules and interstitial tissue. Hypertrophy and hyperplasia were not observed in heart cardiomyocytes and rhabdomyocytes of gastrocnemius muscle. The lung sections displayed a normal appearance with normal bronchiole and alveolus. Histologic alterations, including increased vesicle size in the cytoplasm (macrovesiculation), decreased vesicle size in the cytoplasm (microvesiculation) edema, inflammation and dilation of lymphatics and/or venules, were not seen in both brown (BAT) and white fat tissue by TTFD treatment. These results indicated that both white fat and BAT tissues were not targeted by TTFD treatment in each group. Taken together, the histopathological examinations of the organs did not exhibit any pathological changes following TTFD treatments.

## 4. Discussion

In the current study, we used thiamine derivative, thiamine tetrahydrofurfuryl disulfide (TTFD), supplementation to investigate its physiological efficacy on exercise adaption and performance. TTFD supplementation significantly increased the performance of endurance capacities and grip strength without programmed training intervention. In the acute and extended exercise challenges, the biochemical variables related to metabolite and injury indexes, such as lactate, BUN, and CK also significantly improved the benefits of physiological adaption and exercise fatigue recovery. Furthermore, the energy regulation relevant to thiamine functions also demonstrated beneficial effects on energy demands during the exercise duration. In addition, the toxicity of TTFD was assessed after 6 weeks of oral supplementation for safety concerns and it did not show deleterious effects on growth, behavior, clinical variables, body compositions, and histopathology even with relative high dosage supplementations.

In previously reported studies, there has been limited references to investigate thiamine tetrahydrofurfuryl disulfide regarding exercise physiological effects. Endurance performance could be validated for significant improvement with effective doses from 50 mg/kg to 100 mg/kg [19,21]. However, opposite data propose that the TTFD intake did not affect performance of isokinetic force [25] and high-intensity exercise [26]. We believed the supplementation durations and exercise types could be the critical factors to explain the inconsistency and difference. In the current study, TTFD was used in the form of long-term supplementation and was beneficial to aerobic/endurance exercise in the mouse model. Thiamine is needed by nerve cells, or neurons, and supporting nervous system cells, or glia cells, to create neurotransmitters responsible for thoughts, memory and movement. The grip strength could directly reflect neuromuscular health and coordination [27] and thiamine could increase muscular strength possibly via the improvement of neuromuscular fitness (Figure 4).

Exercise related indexes, such like lactate, BUN, CK, and glucose, were widely applied to assess the exercise physiological status. The energy metabolism pathways contained the phosphagen system, glycolysis and the aerobic system, which were initiated at the same time and depended on intensity and duration preference. Therefore, the exercise-induced lactic acid accumulation and the resulting pH decrease in cells have been widely considered to result in fatigue in skeletal muscles [28]. In a previous study, a clinical trial showed that 4 weeks of thiamine supplementation (30 mg/day) could significantly decrease the lactate and ammonia levels during exercise using a bicycle ergometer [29]. However, another report reported on the lactate levels and lactate threshold with high TTFD dose in a 4-day short term supplementation [26]. In the current study, we found that long-term TTFD supplementation (200, and 500 mg/kg, equivalent to human 16 and 40 mg/kg) could significantly decrease exercise-induced lactate levels and improve the lactate production rate (Table 1).

Energy with sufficient supplement is critical to physiological maintenance and energy molecules (ATP, ADP, and AMP) under normal conditions. ATP is consumed by exercise demand and ADP is converted to AMP for ATP replenishment. AMP could be further metabolized to IMP and ammonia by deaminase and the ammonia could be metabolized as BUN via the urea cycle [30]. In the current study, we also found that the blood ammonia index immediately after exercise was significantly lower with TTFD treatments as compared to vehicle (data not shown). Therefore, BUN could be considered as a biomarker for ATP metabolism, not only for kidney function. BUN was significantly decreased with TTFD supplements (Figure 5A) and it reflected the energy sufficient supply from other glycolysis and tricarboxylic acid cycle processes which is consistent with glucose index results (Figure 6). The blood glucose after exercise was significantly elevated in a dose-dependent manner for energy demands and in the recovery phase, glucose could be efficiently metabolized for tissue utilization. A previous report also demonstrated that TTFD could assist glucose metabolism during exercise [21] which is consistent with the thiamine physiological roles in carbohydrate metabolism.

Regular exercise demonstrated many health benefits including a lowered threat of all-cause mortality along with a reduced risk and preventive effects of non-communicable disease. Paradoxically, it is also clear that contracting skeletal muscles generate free radicals and that prolonged and intense exercise can result in oxidative damage to both proteins and lipids in the contracting myocytes [31]. The direct and indirect indexes, including lactate dehydrogenase (LDH), creatine kinase (CK), AST, and ALT, were also used to assess injury risk indexes in previous studies. The extended exercise challenge demonstrated that the CK index was mitigated with dose-dependent TTFD supplements (Figure 5B). Thiamine could inhibit lipid peroxidation and free radical oxidation possibly via the successive transfer of 2H^(+)^ from the NH_(2)_ group of the pyrimidine ring and H^(+)^ from the thiazole ring (after its opening) to reactive substrates [32]. Therefore, TTFD could protect cells from exercise-induced oxidation and indirectly ameliorate the injury index according to the current results. The glycogen content in muscle and liver were not affected by TTFD 100 mg/kg supplementation (Masuda, 2010) in the same species animal model, which is consistent with our data. However, we found that a higher TTFD dose (500 mg/kg) in the long-term could be cause a significant increase in liver and muscular glycogen. TTFD supplement dose and duration seem to be important to glycogen regulation.

The tabulated LD50 levels in mice for thiamine hydrochloride were reported to be 0.07–0.125 g/kg intravenous, 0.317–0.500 g/kg intraperitoneal and 3–15 g/kg orally [33]. In the high-dose thiamine (>600 mg/day) of therapeutic use, the symptoms of fibromyalgia and disease (inflammation and stroke)-induced fatigue could be ameliorated in clinical application [34,35]. However, the toxicity of effective doses with long-term supplementation was not reported in previous studies. We found that TTFD supplementation did not affect the growth, dietary, behaviors, body compositions, and biochemistries (Table 2, Table 3 and Table 4) for the whole duration of the study. Actually, TTFD has higher bioavailability and distribution than thiamine in a variety of organs, especially in the muscle, liver, kidney, and heart [19]. Histological observation which can reveal pathological changes with the long-term and high-dose supplementation demonstrated that the TTFD did not cause or induce organ abnormalities (Figure 8).

## 5. Conclusions

Thiamine is widely acceptable as a pharmaceutical supplement to maintain the physiological function with an RDA dose. In the current study, we proposed that the higher thiamine derivative, TTFD, could significantly improve physical activities and physiological adaption with evidence-based safety validation. For practical application, we recommend that athletes should consume a daily intake of 40 mg/kg TTFD (equivalently converted from mouse 500 mg/kg dose based on body surface area between mice and humans by formula from the US Food and Drug Administration [36]) to improve energy regulation for higher performance in a combined nutritional strategy, including carbohydrate loading for efficient energy demand during extended exercise.

## Figures and Tables

**Table 1:** The effects of TTFD on lactate metabolite profiles during acute exercise challenge.

Time Point	Vehicle	TTFD-1X	TTFD-2X	TTFD-5X
	Lactate (mmol/L)			
Before swimming (A)	2.4 ± 0.2 ^a^	2.4 ± 0.3 ^a^	2.3 ± 0.2 ^a^	2.4 ± 0.1 ^a^
After swimming (B)	6.1 ± 0.3 ^b^	4.9 ± 0.4 ^a^	4.5 ± 0.3 ^a^	4.2 ± 0.2 ^a^
After a 20 min rest(C)	4.1 ± 0.3 ^b^	3.6 ± 0.2 ^ab^	3.2 ± 0.2 ^a^	3.0 ± 0.1 ^a^
	Rate of lactate production and clearance			
Production rate = B/A	2.96 ± 0.2 ^b^	2.49 ± 0.5 ^ab^	2.06 ± 0.3 ^a^	1.82 ± 0.1 ^a^
Clearance rate = (B-C)/B	0.31 ± 0.1	0.26 ± 0.1	0.26 ± 0.1	0.26 ± 0.01

The lactate metabolites were assessed for the four groups allocated as vehicle, TTFD-1X, TTFD-2X, and TTFD-5X at three repeated time points within group. Lactate production rate was calculated as, after exercise divided by before exercise (B/A) and the lactate difference between after exercise and after rest divided by after rest was defined as the clearance rate. Values in the same row with different superscript letters (a, b) differ significantly, *P* < 0.05, by one-way ANOVA.

**Table 2:** Growth curve and dietary profiles during experiments.

Time Point	Vehicle	TTFD-1X	TTFD-2X	TTFD-5X
Initial BW (g)	31.8 ± 0.2	32.2 ± 0.3	31.7 ± 0.3	32.0 ± 0.3
1st wk BW (g)	32.3 ± 0.3	32.4 ± 0.3	32.2 ± 0.2	32.1 ± 0.2
2nd wk BW (g)	34.5 ± 0.3	34.5 ± 0.5	34.0 ± 0.3	34.0 ± 0.4
3rd wk BW (g)	36.5 ± 0.4	36.2 ± 0.7	36.2 ± 0.5	36.0 ± 0.5
4th wk BW (g)	37.7 ± 0.4	37.0 ± 0.8	37.4 ± 0.6	36.8 ± 0.6
5th wk BW (g)	38.1 ± 0.7	37.5 ± 0.7	37.1 ± 0.5	37.1 ± 0.6
6th wk BW (g)	38.5 ± 0.7	37.5 ± 0.7	38.2 ± 0.5	37.5 ± 0.6
Final BW (g)	38.4 ± 0.8	37.4 ± 0.8	38.1 ± 0.5	37.6 ± 0.5
Water intake (mL/mouse/day)	9.1 ± 0.2	9.1 ± 0.2	9.0 ± 0.2	8.9 ± 0.2
Chow 5001 (g/mouse/day)	7.0 ± 0.1	6.9 ± 0.1	6.9 ± 0.1	6.9 ± 0.1

Weight and diet were measured regularly for the four groups allocated as vehicle, TTF D-1X, TTFD-2X, and TTFD-5X. All the data are represented as mean ± SEM and analyzed by one-way ANOVA.

**Table 3:** Effects of TTFD on body compositions.

Characteristic	Vehicle	TTFD-1X	TTFD-2X	TTFD-5X
Liver (g)	2.17 ± 0.15	2.18 ± 0.10	2.19 ± 0.06	2.19 ± 0.04
Muscle (g)	0.38 ± 0.01	0.39 ± 0.01	0.39 ± 0.01	0.39 ± 0.01
Kidney (g)	0.62 ± 0.01	0.60 ± 0.02	0.60 ± 0.02	0.61 ± 0.01
Heart (g)	0.19 ± 0.01	0.19 ± 0.01	0.19 ± 0.01	0.19 ± 0.01
Lung (g)	0.26 ± 0.01	0.26 ± 0.01	0.26 ± 0.01	0.26 ± 0.01
EFP (g)	0.31 ± 0.03	0.30 ± 0.02	0.26 ± 0.03	0.27 ± 0.03
BAT (g)	0.12 ± 0.01	0.12 ± 0.01	0.12 ± 0.01	0.12 ± 0.01
Relative liver weight (%)	5.63 ± 0.34	5.80 ± 0.19	5.75 ± 0.15	5.83 ± 0.09
Relative muscle weight (%)	1.00 ± 0.04	1.05 ± 0.04	1.01 ± 0.02	1.04 ± 0.02
Relative kidney weight (%)	1.62 ± 0.03	1.60 ± 0.04	1.58 ± 0.03	1.63 ± 0.05
Relative heart weight (%)	0.51 ± 0.01	0.52 ± 0.02	0.50 ± 0.02	0.52 ± 0.03
Relative lung weight (%)	0.68 ± 0.02	0.68 ± 0.01	0.68 ± 0.01	0.68 ± 0.02
Relative EFP weight (%)	0.82 ± 0.08	0.81 ± 0.06	0.69 ± 0.08	0.70 ± 0.06
Relative BAT weight (%)	0.31 ± 0.02	0.31 ± 0.01	0.30 ± 0.01	0.33 ± 0.02

Data are mean ± SEM for *n* = 10 mice in each group. EFP: epididymal fat pad; BAT: brown adipose tissue.

**Table 4:** The effects of TTFD on clinical biochemical analysis at the end of the experiment.

Parameter	Vehicle	TTFD-1X	TTFD-2X	TTFD-5X
AST(U/L)	77 ± 3	77 ± 3	77 ± 4	77 ± 3
ALT(U/L)	43 ± 1	41± 1	42 ± 1	42 ± 2
NH3 (µmol/L)	219 ± 7	216 ± 10	215 ± 11	212 ± 10
CK(U/L)	194 ± 22	189 ± 19	171 ± 14	179 ± 18
GLU(mg/dL)	152 ± 8	147 ± 11	146 ± 10	149 ± 8
CREA(mg/dL)	0.4 ± 0.001	0.4 ± 0.001	0.4 ± 0.001	0.4 ± 0.001
BUN (mg/dL)	23.3 ± 0.6	23.2 ± 0.4	22.4 ± 0.5	22.3 ± 0.7
UA (mg/dL)	1.5 ± 0.1	1.6 ± 0.2	1.5 ± 0.1	1.6 ± 0.2
TC (mg/dL)	142 ± 1 ^b^	137 ± 3 ^ab^	134 ± 1 ^a^	135 ± 2 ^a^
TG (mg/dL)	180 ± 7	170 ± 7	163 ± 8	165 ± 9
ALB(g/dL)	2.6 ± 0.1	2.7 ± 0.1	2.7 ± 0.1	2.7 ± 0.1
TP(g/dL)	5.2 ± 0.1	5.3 ± 0.1	5.3 ± 0.1	5.3 ± 0.1

Data are mean ± SEM for *n* = 10 mice in each group. Values in the same row with different superscript letters (a, b) differ significantly, *P* < 0.05, by one-way ANOVA; AST, aspartate aminotransferase; ALT, alanine transaminase; NH_3_, Ammonia; CK, Creatine kinase; GLU, glucose; CREA, Creatinine; BUN, blood urea nitrogen; UA, uric acid; TC, total cholesterol; TG, triacylglycerol; ALB, albumin; TP, total protein.
